# Postmenopausal hormone therapy and the risk of rheumatoid arthritis: results from the Swedish EIRA population-based case-control study

**DOI:** 10.1007/s10654-015-0004-y

**Published:** 2015-03-12

**Authors:** Cecilia Orellana, Saedis Saevarsdottir, Lars Klareskog, Elizabeth W. Karlson, Lars Alfredsson, Camilla Bengtsson

**Affiliations:** 1 Institute of Environmental Medicine, Karolinska Institutet, Stockholm, Sweden; 2 Rheumatology Unit, Department of Medicine, Karolinska University Hospital, Karolinska Institutet, Stockholm, Sweden; 3 Brigham and Women’s Hospital, Harvard Medical School and Harvard School of Public Health, Boston, MA USA; 4 Center for Occupational and Environmental Medicine, Stockholm County Council, Stockholm, Sweden

**Keywords:** Rheumatoid arthritis, Postmenopausal hormone therapy, Antibodies to citrullinated peptides (ACPA), Etiology, Epidemiology

## Abstract

To study the association between postmenopausal hormone therapy (PMH) use and the risk of rheumatoid arthritis (RA) stratifying the cases by the presence/absence of antibodies against citrullinated peptides (ACPA). A subset of the Epidemiological Investigation of RA (EIRA), a population-based case-control study, comprising postmenopausal women aged 50–70 living in Sweden, between 2006 and 2011 was analysed (523 cases and 1057 controls). All participants answered an extensive questionnaire, including questions regarding PMH use and potential confounders (education, smoking, BMI, oral contraceptives, reproductive factors). We calculated odds ratios (OR) of developing ACPA-positive/-negative RA, with 95 % confidence intervals (CI) and adjusted for age, residential area and smoking. Current users of PMH had a decreased risk of ACPA-positive RA compared with never users (OR 0.6, 95 % CI 0.3–0.9). The decreased risk was observed mainly in the age-group 50–59 years (OR 0.3, 95 % CI 0.1–0.8) but not in the age-group 60–70 years (OR 0.8, 95 % CI 0.4–1.4). Among current users of a combined therapy (estrogen plus progestogens) an OR of 0.3 (95 % CI 0.1–0.7) of ACPA-positive RA was observed, while no significant association was found among women who used estrogen only (OR 0.8, 95 % CI 0.5–1.6). No association between PMH use and ACPA-negative RA was found. PMH use might reduce the risk of ACPA-positive RA in post-menopausal women over 50 years of age, but not of ACPA-negative RA. The negative influence of this treatment on the risk of other chronic conditions cannot be overlooked.

## Introduction

Rheumatoid arthritis (RA) is among the most common autoimmune diseases, a criterium based syndrome characterized by chronic inflammation in joints, with a multifactorial etiology [1, 2]. The disease is 2–3 times more common among women, where the estimated disease prevalence is 2–2.7 % in the age group above 60 years [3]. A higher incidence of RA is seen among women compared to men across all ages, [4–6] and the highest incidence among women has been reported between 55 and 64 years of age, during the peri- or postmenopausal stage, [4, 6] however one study has reported a later peak [7]. Hormonal factors, such as estrogen, have been hypothesized to be of importance for disease development. [8–18].

The use of postmenopausal hormone (PMH) therapy for menopause related symptoms in relation to RA risk has been explored in several studies, most of them showing no association [12, 13, 19–26] while a few have reported an increased [27] or decreased risk of developing RA [28, 29]. One report has indicated that the use of PMH among women carrying the HLA-DRB1 shared epitope (SE) alleles may protect against the development of criterium-defined RA in a population of women with early undifferentiated arthritis, and that this prevention is associated with a reduction of antibodies to citrullinated peptides (ACPA) [28]. Nevertheless, to the best of our knowledge, no study has investigated the association between PMH use and the risk of ACPA-positive as compared to ACPA-negative RA in a setting where exposure to PMH was ascertained in a healthy population.

Emerging evidence supports that RA consists of two subsets characterized by the presence or absence of ACPA, with different causes and severity of disease course. The majority of all cases (around two-thirds) are ACPA-positive with no major differences between men and women, but whether the high incidence among early postmenopausal women mainly is represented by ACPA-positive cases has to our knowledge not been reported. For ACPA-positive RA several risk factors have been identified, including smoking, the PTPN22*R620W risk allele, and the HLA-DRB1 SE allele [2, 30–33]. In contrast, few risk factors have been identified for the ACPA-negative subgroup of RA [1, 2].

The aim of the present report was to investigate the association between PMH use among postmenopausal women and the risk of developing RA stratifying the cases by ACPA status (positive/negative).

## Methods

### Study design

This study is based on a subset of the Swedish population based case-control study, named Epidemiological Investigation of RA (EIRA), comprising postmenopausal women aged 50–70 years living in defined geographical parts of Sweden, recruited between 2006 and 2011. The general design of EIRA has been described in detail elsewhere [34]. Incident cases of RA were included (81 % were diagnosed with RA within 1 year of symptom onset) and diagnosed by rheumatologists according to the American College of Rheumatology 1987 criteria for RA [35]. One case was only diagnosed according to the new criteria from 2010 [36]. Two female controls per case were randomly selected from the national population register, matched to the case by age, and residential area. If a selected control was not reached or denied participation, another control was invited to participate.

### Data collection

Cases and controls completed an extensive questionnaire regarding life-style and environmental exposures. The questions regarding PMH use included the type of medication and the time (years) of initiation and end of the therapy. Medications were later coded according to the Anatomical Therapeutic Chemical (ATC) classification system [37] and classified as estrogen only or a combination of estrogen plus progestogen. The latter group represents a broad classification including the natural hormone, progesterone, and the synthetic form, progestin [38] and included both combined and sequential regimens.

Potential confounders collected through the questionnaire included parity, use of oral contraceptives, age of menopause, age of menarche, age at first birth, smoking habits, height and weight (to calculate BMI), and education. History of cancer and cardiovascular/circulatory conditions previously diagnosed by a physician were also reported through the questionnaire and coded according to the International Classification of Diseases (ICD-10).

In the age-group 50–70 we identified 568 cases and invited 1409 controls (of whom not all might have been post-menopausal), 552 (97 %) cases and 1143 (81 %) controls answered the questionnaire. In total, 523 participating cases and 1057 controls reported themselves as post-menopausal. Blood samples were available from all participating cases.

### Antibody assays

The blood samples were assayed for ACPA-status using the Immunoscan-RA Mark2 ELISA test (Euro-Diagnostica, Malmo, Sweden) [39, 40]. The cut-off is 25 U/ml for ACPA-positive RA. Five cases that lacked information on ACPA-status were excluded from the analyses.

### Assessment of exposure

The year when the first symptoms of RA occurred was defined as the index-year for each case. Controls were then assigned the same index-year as their matched case.

Women were considered postmenopausal if they replied ‘yes’ to the question: ‘Has your menstruation ceased?’. A total of 13 cases and 31 controls were excluded from the analysis since they did not provide age of menopause. Women whose menopause occurred during or after the index-year (25 cases and 56 controls) were also excluded from the analyses.

Current users of PMH were defined as those women who were using PMH during the index-year and who initiated PMH use prior to onset of symptoms. Past users of PMH were defined as those women who had ceased using hormones at least 1 year before the index-year. Ever users were defined as current and past users while never users correspond to women who had never used PMH before the index-year. Two cases and seven controls were excluded at this stage since they started using hormone therapy during the index-year.

Postmenopausal women who reported use of progestogens alone (not in combination with estrogen) were excluded from the analyses (11 cases and 28 controls), since their use has other medical indications than menopausal symptoms [41].

Written informed consent was given by all participants and ethical approval was obtained from the Regional Ethical Review Board at Karolinska Institutet, Stockholm, Sweden.

### Statistical analyses

Odds ratios (OR) with 95 % confidence intervals (CI) were calculated for ACPA-positive and ACPA-negative RA, associated with ever, current and past use of PMH. Postmenopausal women who never used PMH were used as the reference group. We conducted both unmatched and matched analyses (unconditional/conditional logistic regression), but we only present unconditional results since they were in close agreement with the conditional analyses, but had a higher precision.

Duration of use was categorized according to the median value among the controls (1–6 and ≥7 years).

We adjusted for the matching variables (age and residential area) and smoking (pack-years). Additional adjustments for parity (yes/no), number of children (1, 2, 3 and ≥4), body mass index (BMI <25/≥25), use of oral contraceptives (ever/never), breastfeeding (≤6, 7–10, 11–16 and >16 months in total), age of menarche (≤11, 12, 13, ≥14 years), age (years) at first birth (<21, 21–24, 25–28, >28), years between last delivered child and the index-year (0–24, 25–30, 31–37, >37), and formal education (university level, yes/no) did not substantially change the ORs and were therefore not retained in the final analyses.

We performed separate analyses excluding cases and controls with history of breast cancer (19 cases and 50 controls) and cardiovascular conditions (acute myocardial infarction, angina, stroke and embolism and thrombosis; 20 cases and 48 controls). These analyses did not change our results and we therefore show results without these exclusions.

All analyses were carried out using the statistical analysis system (SAS) version 9.2.

## Results

In total, 467 cases and 935 controls were included in the analyses. In all, 303 (64.9 %) cases were ACPA-positive and the mean duration of disease at inclusion in the study was 10 months for both ACPA-positive and ACPA-negative RA. Cases were more likely to be ever smokers, overweight, and to have a lower educational level (Table 1).

**Table 1:** Characteristics of participating cases and controls, postmenopausal women aged 50–70

	Cases (n = 467)		Controls (n = 935)
	ACPA-positive RA 303 (64.9 %)	ACPA-negative RA 164 (35.1 %)	
Age at inclusion, mean (SD), (years)^a^	60.8 (4.8)	60.6 (5.0)	61.0 (4.9)
Age at menarche (years), mean (SD)	13.3 (1.4)	13.2 (1.5)	13.2 (1.5)
Age at menopause, mean (SD)	50.1 (5.0)	50.0 (5.3)	50.3 (4.9)
PMH use^b^			
Current user	22 (7.3)	18 (11.0)	105 (11.2)
Past user	68 (22.4)	37 (22.6)	197 (21.1)
Never users	209 (69.0)	109 (66.4)	626 (67.0)
Missing	4 (1.3)	0 (0)	5 (0.5)
Type of PMH			
Estrogen	57 (63.3)	31 (56.4)	165 (54.3)
Estrogen + progestogens	33 (36.7)	24 (43.6)	139 (45.7)
Parous	258 (85.2)	139 (84.8)	814 (87.1)
Age at first birth, mean (SD)	23.9 (4.6)	24.6 (5.0)	24.8 (4.7)
Number of pregnancies	2.2 (1.0)	2.2 (0.8)	2.2 (0.8)
Ever use of OCP	203 (67.0)	108 (65.9)	642 (68.7)
Ever smoker	228 (75.8)	114 (70.4)	503 (54.3)^c^
BMI ≥ 25	153 (50.8)	87 (53.1)	423 (45.5)^d^
University degree	71 (23.4)	52 (31.7)	312 (33.4)^d^

EIRA, Sweden, 2006–2011

Values are numbers (percentages) unless otherwise stated

*ACPA* antibodies to citrullinated peptides antigens, *RA* rheumatoid arthritis

^a^Index-year for the controls obtained from their matched case

^b^Two controls (0.2 %) had only information on year of initiation and type of therapy and they were defined as ever users. Missing information on PMH use for 4 cases (all ACPA-positive RA) and 5 controls

^c^ *p* value < 0.0001 for the difference between cases and controls

^d^ *p* value < 0.05 for the difference between cases and controls

### Current/past use of PMH and risk of ACPA-positive/-negative RA

Current users of PMH had a decreased risk of developing ACPA-positive RA compared with never users (OR 0.6, 95 % CI 0.3–0.9) in the adjusted model, but no association was found for past users. There was no association between ever, current or past users of PMH and the risk of developing ACPA-negative RA (Table 2).

**Table 2:** Odds ratio of ACPA-positive RA, ACPA-negative RA and RA overall according to ever, current and past use of PMH among women aged 50–70

ACPA status	Use of PMH	Ca/Co	OR 95 % CI^a^	OR 95 % CI^b^
ACPA-positive	Ever^c^	90/304	0.9 (0.7–1.2)	0.9 (0.6–1.2)
	Current	22/105	0.6 (0.4–1.0)	0.6 (0.3–0.9)
	Past	68/197	1.0 (0.7–1.4)	1.1 (0.8–1.5)
	Never	209/626	1.0	1.0
	Missing^d^	4/5	–	–
ACPA-negative	Ever	55/304	1.1 (0.7–1.5)	1.0 (0.7–1.4)
	Current	18/105	1.0 (0.6–1.7)	0.9 (0.5–1.5)
	Past	37/197	1.1 (0.7–1.7)	1.1 (0.7–1.7)
	Never	109/626	1.0	1.0
	Missing^d^	0/5	–	–
RA overall	Ever	145/304	0.9 (0.7–1.2)	0.9 (0.7–1.2)
	Current	40/105	0.7 (0.5–1.1)	0.7 (0.4–1.0)
	Past	105/197	1.1 (0.8–1.4)	1.1 (0.8–1.4)
	Never	318/626	1.0	1.0
	Missing^d^	4/5	–	–

EIRA, Sweden, 2006–2011

*ACPA* antibodies to citrullinated peptides antigens, *PMH* postmenopausal hormone, *RA* rheumatoid arthritis, *Ca/Co* number of cases/controls, *OR* odds ratio, *CI* confidence interval

^a^Adjusted by age and residential area

^b^Adjusted by age, residential area and smoking (pack-years)

^c^Two controls had only information on year of initiation and type of therapy and they were defined as ever users

^d^Missing information on PMH use

### Duration of PMH and risk of ACPA-positive/-negative RA

A shorter duration of PMH use (1–6 years) was associated with a decreased risk of ACPA-positive RA among current users (adjusted OR 0.3, 95 % CI 0.1–0.7), while the association was not statistically significant for the ACPA-negative subset (adjusted OR 0.4, 95 % CI 0.1–1.3). A longer duration of PMH among current as well as past users was not associated with ACPA-positive RA. A longer duration was associated with a non-significantly increased risk of ACPA-negative RA among current (OR 1.3, 95 % CI 0.7–2.4) but not among past PMH users (OR 0.9, 95 % CI 0.5–1.7) (Table 3).

**Table 3:** Odds ratio of ACPA-positive RA, ACPA-negative RA according to duration of use among current and past PMH users, women aged 50–70

Duration of PMH use (years)^a^	ACPA-status	PMH use	Ca/Co	OR 95 % CI^b^	OR 95 % CI^c^
1–6 years	ACPA-positive	Ever	38/147	0.8 (0.5–1.1)	0.8 (0.5–1.2)
		Current	4/44	0.3 (0.1–0.7)	0.3 (0.1–0.7)^d^
		Past	34/103	1.0 (0.6–1.5)	1.0 (0.7–1.6)
		Never	209/626	1.0	1.0
	ACPA-negative	Ever	25/147	1.0 (0.6–1.6)	0.9 (0.6–1.5)
		Current	3/44	0.4 (0.1–1.3)	0.4 (0.1–1.3)
		Past	22/103	1.2 (0.7–2.1)	1.2 (0.7–2.0)
		Never	109/626	1.0	1.0
7 years or more	ACPA-positive	Ever	52/152	1.0 (0.7–1.5)	1.0 (0.7–1.5)
		Current	18/59	0.9 (0.5–1.6)	0.8 (0.5–1.5)
		Past	34/93	1.1 (0.7–1.7)	1.1 (0.7–1.8)
		Never	209/626	1.0	1.0
	ACPA-negative	Ever	29/152	1.2 (0.7–1.8)	1.1 (0.7–1.7)
		Current	15/59	1.5 (0.8–2.7)	1.3 (0.7–2.4)
		Past	14/93	0.9 (0.5–1.7)	0.9 (0.5–1.7)
		Never	109/626	1.0	1.0

EIRA, Sweden, 2006–2011

*ACPA* antibodies to citrullinated peptides antigens, *RA* rheumatoid arthritis, *PMH* postmenopausal hormone, *Ca/Co* number of cases/controls, *OR* odds ratio, *CI* confidence interval

^a^Duration of PMH use among those with available information

^b^Adjusted by age and residential area

^c^Adjusted by age, residential area and smoking (pack-years)

^d^ *p* = 0.0095

### Current/past use of PMH and risk of ACPA-positive/-negative RA in different age groups

The decreased risk of ACPA-positive RA among current users of PMH was observed mainly in the age group 50–59 years (OR 0.3, 95 % CI 0.1–0.8), while no significant effect was observed in the age group 60–70 years (OR 0.8, 95 % CI 0.4–1.4). No association between past PMH use and the risk of ACPA-positive RA was observed. No association between past/current PMH use and risk of ACPA-negative RA was observed in any of the age categories (Table 4).

**Table 4:** Odds ratio of ACPA-positive, ACPA-negative RA according to ever, current and past use of PMH among different age groups

ACPA status	Use of PMH	50–59 years		60–70 years	
		Ca/Co	OR 95 % CI^a^	Ca/Co	OR 95 % CI^a^
ACPA-positive	Ever	26/95	0.7 (0.4–1.2)	64/209	1.0 (0.7–1.4)
	Current	5/42	0.3 (0.1–0.8)^b^	17/63	0.8 (0.4–1.4)
	Past	21/51	1.1 (0.6–2.0)	47/146	1.1 (0.7–1.6)
	Never	92/266	1.0	117/360	1.0
	Missing^c^	0/0	–	4/5	–
ACPA-negative	Ever	18/95	0.9 (0.5–1.6)	37/209	1.1 (0.7–1.7)
	Current	9/42	0.8 (0.3–2.0)	9/63	0.9 (0.4–1.9)
	Past	9/51	0.9 (0.4–2.1)	28/146	1.2 (0.7–2.0)
	Never	50/266	1.0	59/360	1.0
	Missing^c^	0/0	–	0/5	–

EIRA, Sweden, 2006–2011

*ACPA* antibodies to citrullinated peptides antigens, *RA* rheumatoid arthritis, *PMH* postmenopausal hormone, *Ca/Co* number of cases/controls, *OR* odds ratio, *CI* confidence interval

^a^Adjusted by age, residential area and smoking (pack-years)

^b^ *p* = 0.0138

^c^Missing information on PMH use for four cases (all ACPA-positive RA) and five controls

### Type of therapy and risk of ACPA-positive/-negative RA

Among current users of a combined PMH therapy (estrogen plus progestogens) an OR of 0.3 (95 % CI 0.1–0.7) of developing ACPA-positive RA was observed. There was no significant association between current PMH use and ACPA-positive RA among women who used estrogen only (OR 0.8, 95 % CI 0.5–1.6). For the ACPA-negative subset, no association was found for ever, current or past use of any type of PMH therapy (Table 5).

**Table 5:** Odds ratio of ACPA-positive, ACPA-negative RA according to type of medication, among women aged 50–70

ACPA status	Use of PMH	Estrogen only		Estrogen + progestogens^a^	
		Ca/Co	OR 95 % CI^b^	Ca/Co	OR 95 % CI^b^
ACPA-positive	Ever	57/165	1.1 (0.7–1.5)	33/139	0.7 (0.5–1.1)
	Current	15/50	0.8 (0.5–1.6)	7/55	0.3 (0.1–0.7)^c^
	Past	42/114	1.2 (0.8–1.8)	26/83	1.0 (0.6–1.6)
	Never	209/626	1.0	209/626	1.0
	Missing	4/5	–	4/5	–
ACPA-negative	Ever	31/165	1.0 (0.7–1.6)	24/139	1.0 (0.6–1.6)
	Current	9/50	0.8 (0.3–1.8)	9/55	0.9 (0.4–2.0)
	Past	22/114	1.1 (0.7–1.9)	15/83	1.1 (0.6–2.0)
	Never	109/626	1.0	109/626	1.0
	Missing	0/5	–	0/5	–

EIRA, Sweden, 2006–2011

*ACPA* antibodies to citrullinated peptides antigens, *RA* rheumatoid arthritis, *PMH* postmenopausal hormone, *Ca/Co* number of cases/controls, *OR* odds ratio, *CI* confidence interval

^a^The estrogen plus progestogen group includes both combined and sequential regimens

^b^Adjusted by age, residential area and smoking (pack-years)

^c^ *p* = 0.0078

## Discussion

Our data demonstrate a decreased risk of developing ACPA-positive RA among postmenopausal women who were currently using PMH at onset of their disease. This decreased risk was mainly present among women aged 50–59 years and only among users of a combined therapy of estrogen and progestogens, however the number of individuals were low in several subgroups. We found no association between past PMH use and risk of ACPA-positive RA, or current/past PMH use and the risk of ACPA-negative RA.

Furthermore, we found a decreased risk of ACPA-positive RA among women with a short duration of PMH use. This finding, together with a decreased risk of ACPA-positive RA only among women aged 50–59, might reflect that initiation of PMH relatively close to menopause has an impact on development of ACPA-positive disease. [In line with this reasoning are studies on other conditions (coronary heart disease)] [42]. Due to low number of observations we were not able to disentangle PMH initiation in relation to time of menopause. We were neither able to unravel whether the decreased risk of ACPA-positive RA among those with short duration was conferred to young or older women but the medium duration of PMH use was lower among women aged 50–59 (5 years) than among women aged 60–70 (8 years). Furthermore, according to the low number of observations we were hampered to elucidate the indication of an increased risk of ACPA-negative RA among women with long duration of PMH use.

EIRA is a large population-based case-control study comprising incident cases of RA. To minimize the risk of selection bias, that frequently threats the validity of case-control studies, we selected controls randomly and continuously from the same geographic region as the cases. The participation proportion among controls was 75 % which might introduce such a bias if the controls do not reflect the PMH use in the study base. However, we observed approximately the same frequency of current PMH use among the controls (11 %) as reported in another Swedish study using another study design. According to a prescription register covering the entire population, the PMH use among women aged 50–69 was approximately 9 % during 2007 [43]. Women who started using PMH during the index year were excluded from all analyses, since they might have initiated the treatment after RA onset. However, in a separate analysis inclusion of these women did not alter the results.

We defined postmenopausal women as those who reported cessation of their menses according to self-reported information. There is a potential misclassification given by the lack of detailed information regarding the exact time of absence of menses. We think however that this source of misclassification is non-differential, (i.e. that the cases would recall the absence differently from the controls) and we have minimized this by excluding all women who did not report a specific age of menopause and by restricting our analyses to women aged 50–70 years when the menopause is more likely to have already occurred.

Finally, a major strength of our study was the ability to adjust our results with respect to several potential confounders.

Limitations of our study should be mentioned. The lack of detailed information on the hormonal therapy (i.e. specific types of estrogen and/or progestogens, dose, route of administration) has hindered a more detailed stratification, allowing only a broader classification. Moreover, the overall sample size was relatively small and attenuated when we stratified by other factors of interest, which hampered us from drawing firm conclusions on the associations with age, duration of PMH use and type of medication. Questions might also be raised whether we should adjust for multiple testing. Since our analysis was hypothesis driven we think this is not necessary, but nevertheless, taking multiple comparisons into account by increasing the confidence level to 99 % did not change the results substantially, with significant results for duration and type of preparation, and with borderline significance for age-group analysis. Finally, the reason to use PMH is often vasomotor symptoms (VMS) during the menopausal transition. Unfortunately, we did not have information on VMS to assess whether it is a confounder.

The current knowledge on the association between PMH use and the onset of RA is so far inconclusive. Most of the previous studies have not observed an association [12, 13, 19–26]. Our results are in accordance with a report by Vandenbroucke et al. [29] where current use of substitution hormones was associated with a decreased risk of RA. Our results are also in line with a previous case-control study, where a decreased risk of RA among current users of estrogen plus progestin was found and, as in our study, no association for current users of estrogen only was observed [22]. A similar finding was reported in a nested case-control study, where the current use of hormonal replacement therapy at the time of onset was less likely among the cases [21]. In a more recent study from Salliot et al. [28] the use of hormone replacement therapy in women with early undifferentiated arthritis is proposed to protect against the development of criterium-defined RA in individuals carrying HLA-DRB1 SE alleles (OR 0.43, 95 % CI 0.24–0.77) by reducing the risk for the presence of ACPA. However, to the best of our knowledge, our study is the first one investigating PMH therapy separately for ACPA-positive and ACPA-negative RA, and also exploring the risk according to the type of therapy (estrogen only or estrogen plus progestogens).

According to emerging evidence, ACPA-positive and ACPA-negative RA have different environmental (e.g. smoking), and genetic risk factors (e.g. HLA-DRB1 SE alleles) [2, 30–33]. Thus, our findings of different impact of PMH use, where the reduced risk seems to be restricted to the ACPA positive subset, support the notion of RA as two different disease entities with different/distinct etiology.

Our different result for current users of PMH including only estrogen, compared with users of a combination of estrogens and progestogens, might be explained by an immunomodulatory effect given by the natural hormone progesterone, which has been suggested to differ from the one from estrogens and androgens [44–47]. Moreover, a lower incidence of RA has been described during pregnancy, likely to be due to the anti-inflammatory milieu provided by elevated concentrations of circulating hormones, such as estrogen, corticosteroids and progesterone [48]. More specifically, progesterone is believed to decrease disease activity in the pregnant state, through inhibition of Th1and Th17 pathways and induction of anti-inflammatory molecules [49]. In the postpartum period, when progesterone levels fall to reach normal concentrations, a higher incidence of RA has been reported, [8, 15, 16] finding that has been confirmed by a previous report from our group, but interestingly only confined to ACPA-negative RA [50]. Finally, why these potential mechanisms would act differently in the two subsets of RA remain to be elucidated.

The use of PMH, whether as a single or combination therapy, has been associated with elevated risks of endometrial cancer, breast cancer and cardiovascular diseases, among other conditions [51, 52]. Inconsistent results with the use of different therapy regimens have led to the notion of the absence of a group effect, especially when it comes to progestogens [38], however PMH initiation in relation to time of menopause might be important [42]. Although we were only able to perform analyses for broad types of medication, we consider our study as a first approach in disentangling the role of PMH in the etiology of RA.

In summary, we found a decreased risk of developing ACPA-positive RA among postmenopausal women who were currently using PMH at the time of onset of the disease. Although our results indicate a protective effect of PMH therapy in the development of RA, the negative influence of this treatment on the risk of developing other chronic conditions cannot be overlooked. Further research is required to explore the biological mechanisms behind our findings but our results contribute to the knowledge of hormonal risk factors, such as the use of PMH, and their impact on the subgroups of RA.
